# Cerebrospinal fluid promotes survival and astroglial differentiation of adult human neural progenitor cells but inhibits proliferation and neuronal differentiation

**DOI:** 10.1186/1471-2202-11-48

**Published:** 2010-04-08

**Authors:** Judith Buddensiek, Alexander Dressel, Michael Kowalski, Uwe Runge, Henry Schroeder, Andreas Hermann, Matthias Kirsch, Alexander Storch, Michael Sabolek

**Affiliations:** 1 Department of Neurology, Ernst Moritz Arndt University of Greifswald, 17475 Greifswald, Germany; 2 Department of Neurosurgery, Ernst Moritz Arndt University of Greifswald, 17475 Greifswald, Germany; 3 Department of Neurology and Centre for Regenerative Therapies Dresden (CRTD), Dresden University of Technology, 01307 Dresden, Germany; 4 Department of Neurosurgery, Dresden University of Technology, 01307 Dresden, Germany

## Abstract

**Background:**

Neural stem cells (NSCs) are a promising source for cell replacement therapies for neurological diseases. Growing evidence suggests an important role of cerebrospinal fluid (CSF) not only on neuroectodermal cells during brain development but also on the survival, proliferation and fate specification of NSCs in the adult brain. Existing *in vitro *studies focused on embryonic cell lines and embryonic CSF. We therefore studied the effects of adult human leptomeningeal CSF on the behaviour of adult human NSCs (ahNSCs).

**Results:**

Adult CSF increased the survival rate of adult human NSCs compared to standard serum free culture media during both stem cell maintenance and differentiation. The presence of CSF promoted differentiation of NSCs leading to a faster loss of their self-renewal capacity as it is measured by the proliferation markers Ki67 and BrdU and stronger cell extension outgrowth with longer and more cell extensions per cell. After differentiation in CSF, we found a larger number of GFAP^+ ^astroglial cells compared to differentiation in standard culture media and a lower number of β-tubulin III^+ ^neuronal cells.

**Conclusions:**

Our data demonstrate that adult human leptomeningeal CSF creates a beneficial environment for the survival and differentiation of adult human NSCs. Adult CSF is *in vitro *a strong glial differentiation stimulus and leads to a rapid loss of stem cell potential.

## Background

Neural stem cells (NSCs) are a promising source for cell replacement therapies in the brain and the spinal cord [1]. It is common knowledge that NSCs can be extracted from fetal brain [2-4] or generated from embryonic stem cells [2,5-8]. Furthermore, NSCs can also be isolated from different regions of the adult brain such as the hippocampus and the subventricular zone and from some non-neurogenic regions such as the spinal cord [2,3,9-13] or the periventricular regions of the whole neuroaxis [14]. NSCs are able to replicate and generate all neuroectodermal lineages, namely neurons, astroglia and oligodendroglia [2,3,15]. During *in vitro *expansion, NSCs grow in so-called "neurospheres" or adherent cultures. Neurospheres are either multicellular aggregates or clones originating from one single cell depending on the cell density [3,9,11,15]. In previous studies, the isolation and successful long-term expansion of human NSCs from the adult hippocampus, the adult olfactory bulb and adult post-mortem tissues have been reported [16-20]. In these studies, ahNSCs have successfully been expanded for more than 30 population doublings using serum-free culture medium which is normally supplemented with the mitogenes epidermal growth factor (EGF) and fibroblast growth factor 2 (FGF-2). Removal of the mitogenes leads to spontaneous differentiation of adult NSCs into neurons, astroglia and oligondendroglia [16-20].

Investigations examining the influence of CSF and its contents on survival, proliferation and differentiation of NSCs were so far mostly performed with embryonic avian CSF [21-23]. In these investigations, NSCs cultured in CSF showed an increased survival rate and a higher number of BrdU positive, DNA synthesizing nuclei, compared to standard culture media. In addition a larger number of beta-tubulin III^+^, immature neurons, has been detected compared to standard culture media, indicating a positive influence of embryonic avian CSF on neurogenesis. As there are well known differences between avian and mammalian CSF [24] with a much greater complexity of mammalian CSF, the transferability of these results to human conditions is not yet clear. Also, they might be a difference in between effects of adult CSF on NSCs and embryonic CSF effects. Still, this is not yet investigated, even though it has been suggested that CSF plays an important role not only during brain development but also for the survival, proliferation and differentiation of neuroectodermal stem cells *in vivo *[21-23,25-27]. In this present study we therefore used an *in vitro *cell system of adult human NSCs in culture as a model to investigate the effects of adult human CSF on NSC behaviour.

## Results

### CSF increases the survival rate of adult human NSCs

**Figure 1:**
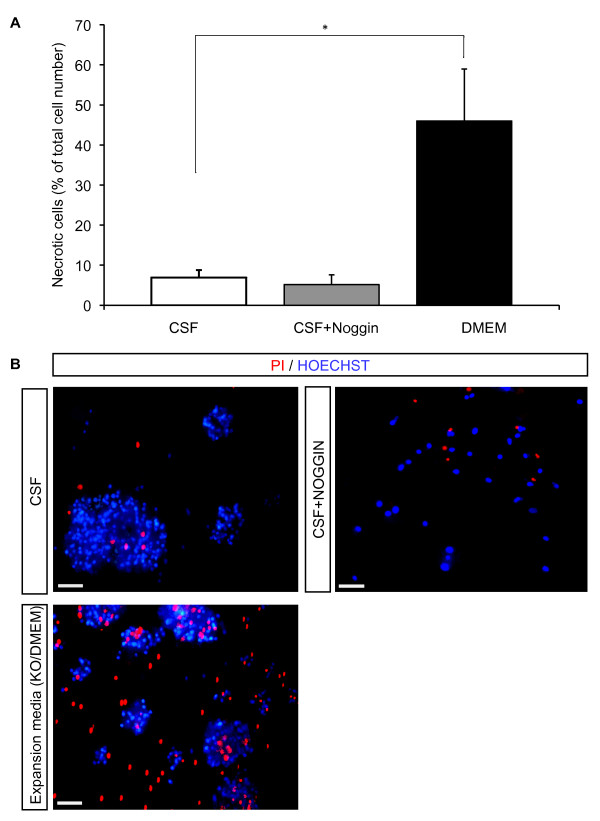
**Survival rate of human adult NSCs during expansion**. **A**, The percentage of necrotic NSCs expanded in culture medium supplemented with EGF and FGF-2 in comparison to CSF with or without 150 ng/ml Noggin was determined 72 hours after starting the expansion process. Values represent the mean ± S.E.M from at least three independent experiments. * indicates *P *< 0.05 and ** *P *< 0.01 compared to each corresponding value in CSF. **B**, Representative microphotographs demonstrating the influence of CSF on survival rate of NSCs. For immunostainings, dead cells were stained with propidiumiodid. Nuclei were counterstained with Hoechst 33342. The upper panel shows NSCs expanded in CSF (left photograph) and CSF+Noggin (right photograph), the lower panel shows NSCs expanded in expansion media supplemented with EGF and FGF. Scale bar = 50 μm

**Figure 2:**
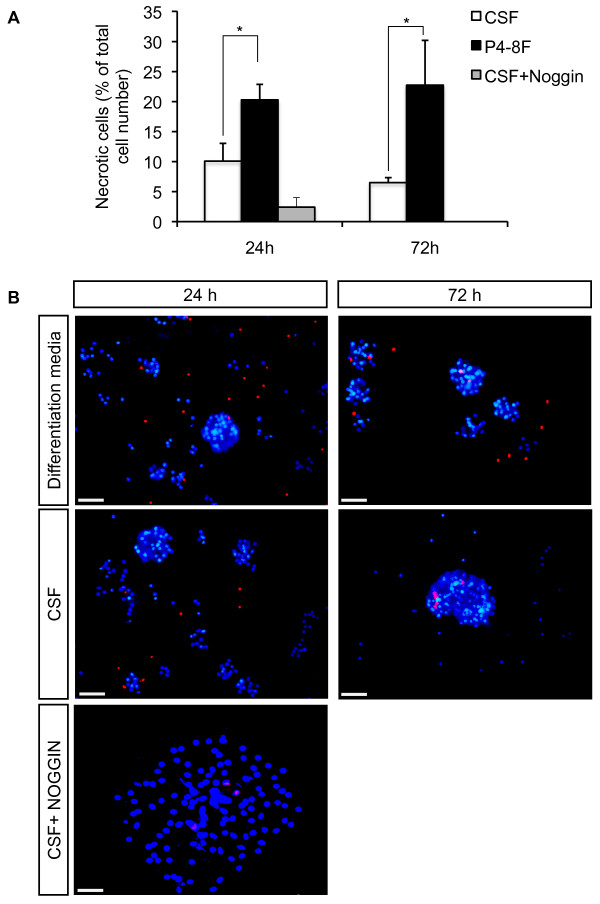
**Survival rate of adult human NSCs during proliferation**. **A**, The percentage of necrotic NSCs was determined at 24 and 72 hours after differentiation on PLL-coated chamber slides in P4-8F or CSF with or without 150 ng/ml Noggin. Values represent the mean ± S.E.M from at least three independent experiments. * indicates *P *< 0.05 and ** *P *< 0.01 compared to each corresponding value in CSF. **B**, Representative microphotographs demonstrating the influence of CSF on the survival rate of NSCs. For immunostainings, dead cells were stained with propidiumiodid. Nuclei were counterstained with Hoechst 33342. The upper panel shows NSCs differentiated in differentiation media P4-8F, the middle panel shows NSCs differentiated in CSF, and the lower panel shows NSCs differentiated in CSF+Noggin. Scale bar = 50 μm

After *in vitro *expansion for at least three passages in standard media, NSCs were transferred to uncoated chamber slides and expanded in standard expansion medium or CSF for additional 72 h. Thereafter, cells were stained with Hoechst 33342 and propidiumiodid to determine the amount of necrotic cells. Our results showed a significantly higher survival rate of NSCs in CSF compared to standard expansion medium (see Materials and Methods) with 6.9 ± 1.9% of necrotic cells in CSF versus 46.0 ± 12.9% in expansion medium (*P *= 0.013; Fig. 1). Addition of the bone morphogenic protein (BMP) inhibitor Noggin did not significantly increase cell death rates compared to CSF without Noggin (5.2 ± 2.4% of necrotic cells, *P *= 0.54; Fig. 1). Similar results were obtained during *in vitro *differentiation with significantly higher survival rates in CSF compared to standard differentiation media: After 24 h in P4-8F media, 20.3 ± 2.6% of NSCs were found necrotic versus 10.1 ± 2.9% in CSF (*P *= 0.021). After 72 h, the proportion was 22.7 ± 7.5% versus 6.5 ± 0.8% for standard media versus CSF, respectively (*P *= 0.038; Fig. 2). Again, addition of Noggin to CSF had no significant influence on the cell death rate during differentiation for the first 24 h (2.4 ± 1.5% of necrotic cells, *P *= 0.09 when compared CSF with CSF plus Noggin condition; Fig. 2).

### CSF inhibits proliferation of adult human NSCs

**Figure 3:**
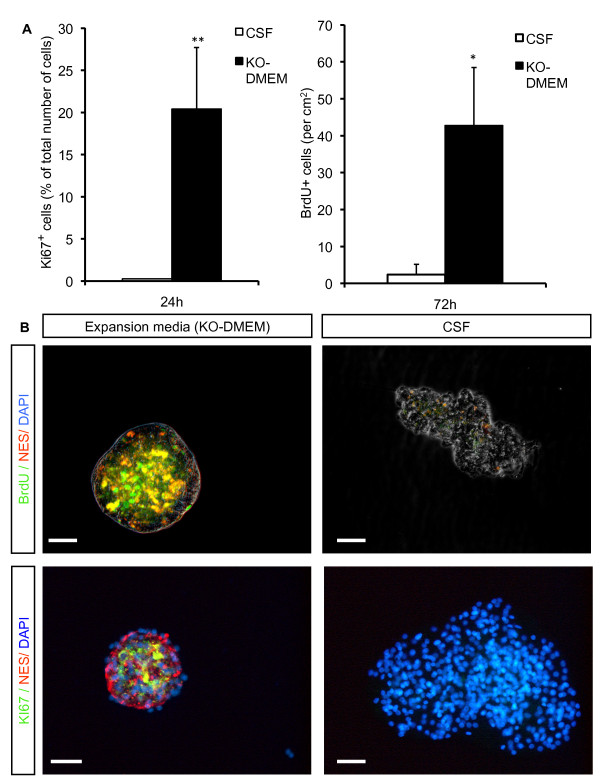
**Investigation of cell proliferation in adult human NSCs during expansion**. The portion of NSCs with potential to self renewal expanded in culture medium supplemented with EGF and FGF-2 or CSF without additional growth factors was determined 24 and 72 hours after medium change. **A**, Percentage of Ki67^+ ^cells after 24 h, number of BrdU^+ ^cells per cm^2 ^after 72 h. Values represent the mean ± S.E.M from at least three independent experiments. *indicates *P *< 0.05 and ** *P *< 0.01 compared to each corresponding value in CSF. **B**, Representative microphotographs demonstrating the influence of CSF on proliferation markers in NSCs. For immunostainings cells were stained with anti-BrdU (upper panel) and anti-Ki67 (lower panel) and anti-Nestin (upper and lower panel). Nuclei were counterstained with DAPI (lower panel). Altered morphology of neurospheres with CSF treatment (right panel) is most likely due to the beginning differentiation process. Scale bar = 50 μm.

To determine the influence of adult human leptomeningeal CSF on self-renewal potential of adult NSCs, we used the proliferation markers Ki67 and BrdU as read-outs. For this, after *in vitro *expansion of at least three passages, cells were transferred to uncoated chamber slides and expanded in culture medium or CSF. The self renewing potential was determined at 24 h (Ki67) and 72 h (BrdU) after media was changed. At each time point, the percentage of Ki67^+ ^and BrdU^+ ^NSCs was significantly higher in expansion medium compared to the one in CSF, representing a larger number of proliferating cells in expansion media. Hence, after 24 h Ki67 was detected in 20.4 ± 7.3% of NSCs expanded in standard medium compared to only 0.2 ± 0.01% of NSCs expanded in CSF (*P *= < 0.001; Fig. 3). For BrdU, we detected 42.8 ± 15.7 BrdU^+ ^cells/cm^2 ^in KO-DMEM, but only 2.4 ± 2.8 BrdU^+ ^cells/cm^2 ^in CSF (*P *= 0.023, Fig. 3).

### CSF increases extension outgrowth velocity from adult human NSCs

**Figure 4:**
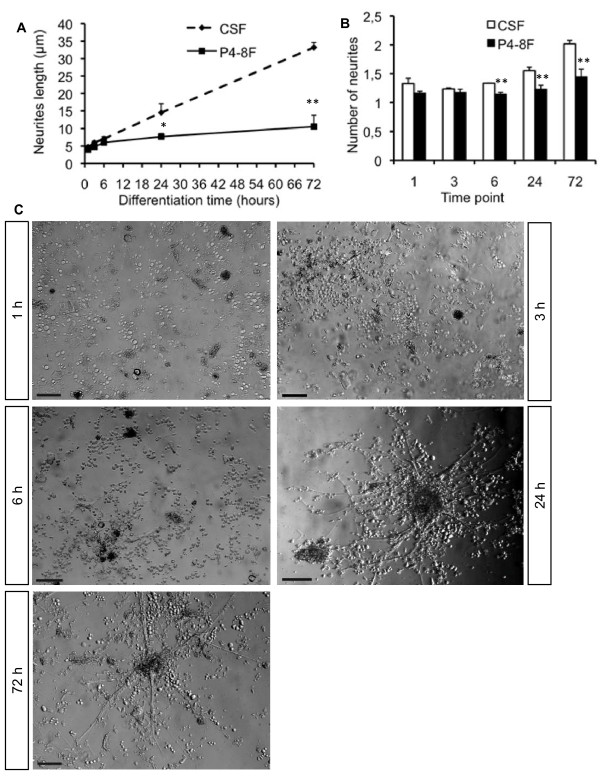
**Cell extension outgrowth in adult human NSCs**. **A**, Length of cell extensions (μm) after 1, 3, 6, 24, 72 hours of differentiation in P4-8F or adult human CSF. **B**, Number of cell extensions at 1, 3, 6, 24, 72 hours of differentiation in P4-8F or human adult CSF. **A and B**, Values represent the mean ± S.E.M from at least three independent experiments. * indicates *P *< 0.05 compared to each corresponding value in CSF. **C**, Representative microphotographs showing cell morphology after 1, 3, 6, 24, 72 hours of differentiation in CSF. Scale bar = 50 μm.

We used the time course of extension outgrowth as a first measure of the differentiation process of NSCs in culture. Thus, following expansion for at least three passages *in vitro *NSCs were allowed to differentiate. Cells were plated onto PLL-coated chamber slides in either differentiation media or CSF. For investigating extension outgrowth, number and length of extensions were determined at 0, 3, 6, 24 and 72 h after starting the differentiation process. Comparing results in P4-8F and CSF, we found significantly longer and a significantly more cell extensions per cell in CSF. After 24 and 72 h the difference was significant as NSC neurites getting twice as long in CSF (mean length: 14.5 ± 2.6 and 33.2 ± 1.4 μm after 24 and 72 h, respectively) as in P4-8F (mean length: 7.7 ± 0.9 and 10.6 ± 3.2; *P *= 0.024 [24 h] and 0.001 [72 h]; Fig. 4). Significant more cell extensions were determined in CSF for the time points 6 h, 24 h and 72 h with an average number of 1.3 ± 0.0 cell extensions in CSF after 6 h compared to 1.2 ± 0.1 neuritis in P4-8F. After 24 h, the mean number of cell extensions was 1.5 ± 0.1 in CSF versus 1.2 ± 0.1 in P48F and after 72 hours cells had at an average of 2.0 ± 0.1 cell extensions in CSF compared to 1.5 ± 0.1 in P4-8F respectively.(*P *= 0.001, 0.008 and 0.005, respectively; Fig. 4).

### CSF facilitates astrogliogenesis but inhibits neurogenesis from adult human NSCs

**Figure 5:**
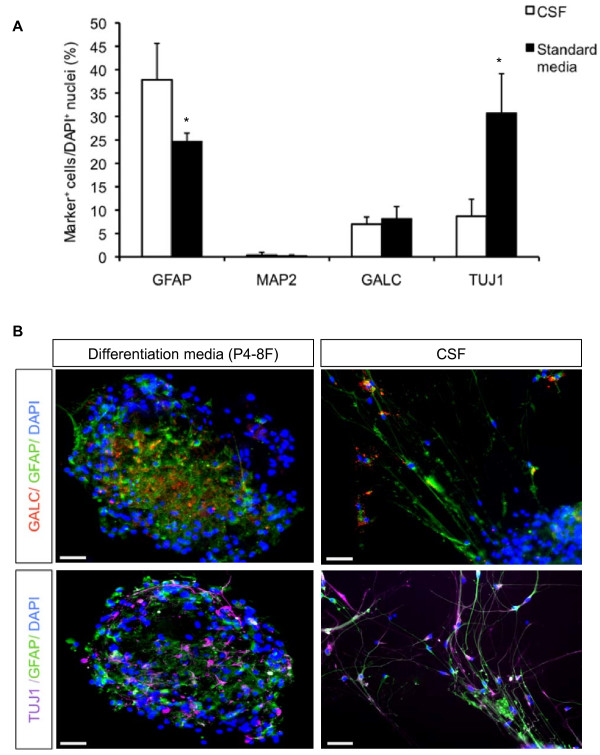
**Neurogenesis and gliogenesis from adult human NSCs**. **A**, The percentage of GFAP^+^, MAP2ab^+^, β-tubulin III and GALC^+ ^cells after differentiation for 10 to 14 days in KO/DMEM or CSF. Results are mean values ± S.E.M from at least three independent experiments. * indicates *P *< 0.05 compared to each corresponding value in CSF. **B**, Representative microphotographs demonstrating the effects of CSF on neuronal and glial differentiation of ahNSCs 10 to 14 days after initiation of the differentiation process. Cells were stained with anti-GFAP, anti-β-tubulin III and anti-GALC. Nuclei were counterstained with DAPI. Scale bar = 50 μm.

To investigate the question whether the presence of adult human leptomeningeal CSF does have an influence on astrogliogenesis, oligodendrogenesis or neurogenesis, adult NSCs were allowed to differentiate for 7 days on PLL-coated chamber slides in either standard differentiation media or CSF. After differentiation in standard media, 24.7 ± 1.7% of the cells were GFAP^+^, in contrast to 37.8 ± 7.8 GFAP^+ ^cells in CSF (*P *= 0.014; Fig. 5). The values for GalC^+ ^oligodendroglial cells and MAP2^+ ^neurons were 8.2 ± 2.6% GalC^+ ^and 0.2 ± 1.7 MAP2^+ ^cells in standard media and 7.0 ± 1.5 GalC^+ ^and 0.4 ± 0.6 MAP2^+ ^cells in CSF (*P *> 0.05). The low percentage of MAP2^+ ^cells in standard media and CSF is most likely caused by the lack of fully differentiated phenotypes, as MAP2 represents a marker for mature neurons. Staining against the neuronal marker protein β-tubulin III, a marker for immature neurons, revealed 8.7 ± 3.6% β-tubulin III^+ ^cells in CSF compared to 30.7 ± 8.4 β-tubulin III^+ ^cells in standard media (*P *= 0.014).

## Discussion

There is growing evidence that CSF plays an important role in physiological as well as pathophysiological processes of the brain including adult neurogenesis [28-30]. In a very recent study of our group, we found adult human leptomeningeal CSF being a promotor of survival, differentiation and astrogliogenesis of fetal NSCs from rat [31]. The influence of CSF on adult NSCs remains however still enigmatic. In this study we therefore used human adult NSCs as an *in vitro *model to study the effects of adult leptomeningeal CSF on NSC behaviour including survival, self renewal and differentiation. The central finding of our study is that *in vitro*, adult CSF promotes survival and differentiation of ahNSCs, but drives the differentiation process towards astrogliogenesis. In accordance with these findings, the loss of stem cell potential is accelerated when cultured in adult CSF. These findings suggest that adult CSF contains key factors involved in the control of cellular proliferation and differentiation processes. This assumption is supported by *in vivo *findings, demonstrating that adult NSCs of the subventricular zone have transitory contact with the ventricular brain cavity and many of them still posses one microcilia which extends into the CSF [32-35]. In addition, it is known from previous studies [21-23] that embryonic CSF has a trophic influence on survival and differentiation of NSCs. However, the loss of stem cell potential is decelerated, which is contradictory to our findings in adult CSF and may explain the abundance of post-mitotic cells in adult CNS.

Based on the knowledge of embryonic CSF studies, it has already been postulated that diffusible factors in embryonic CSF regulate the three basic cellular behavioural parameters of neuroephitelial stem cells and that embryonic CSF may play a key role in brain development *in vivo *[23].

However, how CSF influences neuroectodermal cells during development remains enigmatic, but the components contained in CSF as well as CSF pressure and flow seem to play an important role [26,36]. Regarding the components of CSF influencing neuroectodermal cell behaviour, recent investigations concentrated mainly on proteins, "membranous particles" and amino acids but also on growth factors such as FGF2 [21,24,25,36,37]. In avian and human CSF, many proteins with a known influence on cell survival, neural and glial differentiation, proliferation and signal transduction were found (such as transthyretin, serin, retinol binding protein, heparan sulfate, several apolipoproteins, and FGF2) [22,24,25]. Although it has also been demonstrated that the protein composition of embryonic CSF is more complex than that of adult CSF [22], our results indicate that adult CSF has the capacity to influence the behaviour of adult NSCs in the adult brain, too.

CSF as a beneficial environment for cell survival and growth has also been postulated by *in vivo *studies, investigating for example the behaviour of fetal NSCs after injection in the fourth ventricle of spinal cord lesioned rats with a good survival of grafted cells within the CSF [38,39], as well as in a recent *in vitro *study of our own group with fetal rat NSCs [31]. In the present study, CSF had a general stimulating effect demonstrated by the faster loss of self-renewing capacity and stronger cell extension outgrowth. Adult hNSCs differentiated predominantly into astrocytes (38% in CSF, 25% in standard media) when treated with CSF and to a lower extend into oligodendrocytes and neurons. After differentiation for 7 days, we found 9% of the cells to be β-tubulin^+ ^in CSF. In standard culture media, 31% of the cells were β-tubulin III^+^. Therefore, our data strongly suggest inhibitory effects of CSF on neurogenesis of ahNSCs, but promoting effects on astroliogenesis. Possible factors in CSF influencing differentiation behaviour of NSCs are bone morphogenetic proteins (BMPs): Monoclonal antibodies against BMP7 were for example shown to inhibit CSF induced dendritic outgrowth of neurons. BMP4 was shown to induce neuronal differentiation of NSCs by activating the ERK and inhibiting the GSK3β pathway. Both described effects could be blocked by Noggin, a BMP inhibitor [40,41]. These described effects of BMPs, do have an influence on neuronal differentiation only in fetal NSCs from rat. Until recently, it remained elusive whether these BMPs may also play a role in the observed effects of adult human CSF on astroglial differentiation, but we could show that parts of the CSF mediated effects on fetal rat NSCs could be blocked by Noggin[31]. BMPs thus seem to be at least a part of the soluble factors in the CSF influencing stem cell behaviour in fetal rat NSCs. However, in the present work, we could not find any inhibiting effects of Noggin on CSF survival effects on adult human NSCs. This raises the question whether BMPs influence NSC behaviour only during ontogenesis and therefore have no influence on NSCs derived from the adult brain or whether BMPs might differential effects in human and rat cell systems.

It is well accepted that lumbar CSF is different from ventricular CSF because of both passive diffusion processes across the blood-CSF-barrier and suggested active secretion processes during the cranio-caudal circulation [42]. Consistently, all herein described CSF effects can only be attributed to leptomeningeal CSF. Whether ventricular CSF has similar same effects on NSC behaviour remains elusive. The use of CSF out of ventricular drainages is however problematic, as ventricular drainages are used in patients with obstructed CSF circulation (for example cerebral aqueduct stenosis) or other reasons of elevated brain pressure with defect blood-brain- and blood-CSF-barrier (for example after major stroke, after intracerebral bleedings or inoperable brain tumours). Furthermore, CSF out of ventricular drainages is often contaminated by blood and altered by inflammatory processes, which is why it cannot be used for the examination of healthy CSF effects.

## Conclusions

Together, our results demonstrate that adult leptomeningeal CSF has a trophic influence on adult human NSCs: survival and differentiation into astroglial cells are promoted. Thus, our data point to a pivotal role of CSF in regulating adult neurogenesis under physiological and presumably also under pathological conditions as suggested previously [28-30]. Future experiments are warranted to determine which compounds within the CSF, besides BMPs, might be responsible for the effects on NPC behaviour. It also needs to be investigated whether there are different CSF compounds influencing the behaviour of NSCs originating from fetal or adult brain. This might give us the opportunity to influence resident NSCs of the SVZ to induce their proliferation as well as to promote their migration to pathologically altered brain regions.

## Methods

### Collection of adult human leptomeningeal CSF

The CSF samples were taken for diagnostic purpose from adult patients in the Neurological Clinic of the Ernst Moritz Arndt University of Greifswald by a lumbar puncture. Scientific use of CSF samples was approved by the local Ethics Committee. All patients gave a written informed consent for the diagnostic procedure. Lumbar puncture was performed by standard protocols. The final diagnosis of all patients was Idiopathic Normal Pressure Hydrocephalus (NPH). CSF was only used if all standard parameters were normal (cell count, glucose-, lactate. and albumin-content, immunoglobulin-quotient). Contamination with blood was excluded. In all patients, tumors and infectious diseases were excluded. Surplus CSF from diagnostic samples of all patients was spun down to remove remaining cells, pooled and analyzed to exclude non-sterility and presence of inflammatory markers. The pooled CSF was normal in all standard parameters; contamination with blood was excluded once again. The exact parameters were as follows: Cell count <1/μl, no blood contamination, normal protein of 369 mg/l, normal glucose of 3.6 mmol/l, normal lactate of 2.0 mmol/l, normal ferritine of 7.2 μg/l, normal albumin quotient of 5.0 × 10^-3^, normal immunoglobulin quotients: IgG 2.1 × 10^-3^, IgA 1.1 × 10^-3^, IgM <0.2 × 10^-3^. Pooled CSF samples were aliquoted and frozen at -80° until used.

### Isolation and propagation of adult human neural stem cells (ahNSCs)

Adult human hippocampal tissue was obtained from routine epilepsy surgery procedures (selective amygdalo-hippocampectomia) following informed consent of the patients. All procedures were in accordance with the Helsinki convention and approved by the ethics committees at the EMA University of Greifswald (III UV 77/06) and at Dresden University of Technology (EK47032006). The tissue was stored in ice-cold Hank's balanced salt solution (HBSS) supplemented with 11 mM glucose and 1% penicillin/streptomycin. In all patients, tumours and infectious diseases were excluded by means of high-resolution magnetic resonance imaging and screening for inflammatory markers. Additionally, neuropathological tissue examination did not reveal evidence for tumour formation. For expansion of neurospheres, tissue samples were dissociated using trypsin, DNase and mechanical trituration similar as described previously [19]. Several media and supplements were tested like DMEM, DMEM/F12, Neurobasal (all from Gibco), P4-8F (Athena) with and without N2 or B27 supplements (Gibco) and growth factors. Best results concerning especially the amount of primary neurospheres per dissected hippocampal tissue and the early passage propagation was achieved by Knock-Out DMEM medium, supplemented with 10% KO supplement; 0.5 mM glutamine; 1% penicillin/streptomycin (all from Gibco) and 20 ng/ml both EGF and FGF-2 (from Sigma-Aldrich)(KO/DMEM). Therefore this medium was chosen as control expansion medium. Cultures were incubated at 37°C in a humidified atmosphere and lowered O_2 _conditions of 5% CO_2_, 92% N_2 _and 3 ± 2% O_2_. Fresh medium was added once a week, growth factors twice a week. For BrdU labelling, cells were incubated with 10 μm BrdU for 72 h. For immunocytochemistry studies of neurospheres, these were allowed to attach for 2-4 h before been fixed as described below.

### Differentiation conditions

Cells were differentiated by plating them onto poly-L-lysine-coated chamber slides or 6-well-plates in P4-8F (standard differentiation media; from AthenaES, Baltimore, MD, USA, the albumin-content of P4-8F of 250 μg/ml matches the normal albumin content of healthy adult lumbar CSF, the glucose content of P4-8F is in a physiological concentration of 7 mmol/l) or in CSF without adding any growth factors. Some of the differentiation experiments were conducted in the presence of 150 ng/ml recombinant Noggin (R&D System, Minneapolis, MN). For studying cell survival, proliferation and neurite outgrowth cells were allowed to differentiate for up to 72 hours, for investigating cell fate decisions for up to 7 days respectively. Half of the media was changed every third day. For investigating cell morphology, survival rate and marker expression by immunocytochemistry, the cultures were fixed from 0 h up to 72 h and 14 days after starting the differentiation process.

### Immunostainings

For immunocytochemistry, cell cultures were fixed in 4% paraformaldehyde in PBS or with 4% paraformaldehyde/PBS followed by ice-cold acidic ethanol and 2N HCL for BrdU staining. Immuncytochemistry was carried out using standard protocols. Cell nuclei were counter stained with 4,6-diamidino-2-phenylindole (DAPI). To determine the self renewing potential of NPCs, Ki67 expression and BrdU incorporation were used. Ki67 is detected in the nucleus of proliferating cells in all active phases of the cell cycle from the late G1 phase though the M-phase [43,44]. BrdU marks cells within the S-phase of the cell cycle [45]. Antibodies and dilutions were as follows: rabbit anti-glial fibrillary acidic protein (GFAP) polyclonal 1:1000 (Chemicon International, Temecula, CA, USA), mouse anti-microtubuli associated protein (MAP2ab) monoclonal 1:100 (Pharmingen, San Diego, CA, USA), mouse anti-galactocerebroside (GalC) monoclonal 1:500 (Chemicon), rabbit anti-β-tubulin III (Tuj1) monoclonal 1:1000 (Covance, Emeryville, CA, USA), rabbit anti-Ki67 polyclonal 1:500 (Berlin Chemie AG, Berlin, Germany), mouse anti-BrdU 1:16 (Roche Applied Science, Mannheim, Germany) and secondary antibodies conjugated to Alexa 488 and 594 1:500 (Gibco/Invitrogen, Carlsbad, CA, USA).

For determining the survival rate during expansion and differentiation, dead cells were stained with propidiumiodid (PI) 1:50 (Sigma Aldrich, St. Louis, USA), cell nuclei were counter stained with Hoechst 33342 1:1000 (Sigma Aldrich, St. Louis, USA). Images were captured using inverse fluorescence microscopes (DMIL and DMI4000, Leica; Wetzlar, Germany).

### Cell counts, measurements of neurites and statistics

For quantification of the percentage of cells expressing a given marker, the number of positive cells of at least five representative areas per experiment was determined relative to the total number of DAPI/Hoechst-labelled nuclei, or field of view in cm^2 ^where appropriate. Neurite lengths were measured with a semi-automatic distance measurement computer program (VisRoute^®^). In a typical experiment, a total number of 500 to 1,000 cells were counted per marker, and 500 to 1,000 neurites per time point were measured. The mean values of ≥ 3 experiments for each condition are given together with standard deviations. Statistical comparisons were made by ANOVA with post-hoc *t*-test or Dunnett's *t*-test where appropriate. P-values < 0.05 were considered as statistically significant

## Competing interests

The authors declare that they have no competing interests.

## Authors' contributions

JB carried out the experiments and drafted the manuscript. MK and AH participated in part of the experiments. AH helped optimizing the culture conditions. AD provided the CSF samples and supervised the quality control in the CSF laboratory and contributed to the initial study design. HS and MK performed the surgical procedures and provided the tissue samples. UR selected the appropriate patients for the surgical procedures. MS planned the experiments and performed the design and coordination of the study. JB, MS and AS performed the discussion of the study. MS and AS finalized the manuscript. All authors read and approved the final manuscript.
